# Vancomycin dose adjustment in severe burn patients based on trough level for drug effectiveness against pathogens at 1 mg/l minimum inhibitory concentration

**DOI:** 10.1186/cc12645

**Published:** 2013-06-19

**Authors:** JM Silva, AM Oliveira, EV Campos, DS Gomez, MC Ferreira, CS Giraud, CV Silva, SRCJ Santos

**Affiliations:** 1 Burn Unit/Plastic Surgery Division of Clinics Hospital, Medical School, University of Sao Paulo, Butantã, São Paulo, SP, Brazil; 2 School of Pharmaceutical Sciences, University of Sao Paulo, Butantã, São Paulo, SP, Brazil

## Introduction

Vancomycin is usually prescribed to severe burn patients with sepsis from the intensive care burn unit (ICBU) for control of hospital infection. The objective of this study was to evaluate the contribution of dose regimen adjusted in burn patients with renal function preserved based on drug plasma concentration at the trough and pharmacokinetic-pharmacodynamic (PK/PD) correlation.

## Methods

Sixty severe burn patients with documented Gram-positive nosocomial infection from the ICBU were enrolled in a prospective cohort study, and the period of inclusion was 2 years; the protocol was approved by the hospital's ethical committee. Patients of both genders (43 male/17 female) with preserved renal function were investigated (176 sets). The vancomycin dose regimen was initially 2 g daily for the control of infection in burn patients with sepsis. Pharmacotherapeutic follow-up was performed by a serial blood sample collection (2 ml each) for drug plasma measurements [1]. Drug effectiveness was based on the parameter AUC_ss0-24_/MIC >400 [2]; AUC_ss0-24 _was the area under the curve (plasma concentration vs. time) integrated up to 24 hours, and the minimum inhibitory concentration (MIC) from *in vitro *antimicrobial susceptibility testing performed in the hospital [2]. Dose adjustment was required and the daily dose was increased to reach trough levels >10 μg/ml and AUC/MIC >400.

## Results

Characteristics of patients investigated were (mean ± SD): 38.9 ± 14.1 years; 70.0 ± 10.6 kg, 28.0 ± 19.0% total burn surface area. Thermal injury occurred in 51/60 patients, and inhalation injury occurred in 54.9% of them, versus electrical injury reported in 9/60 patients; renal function was monitored by serum creatinine (0.72 ± 0.29 mg/dl) and creatinine clearance (153.7 ± 70.4 ml/minute). Significant increase was chosen by comparison of the initial dose against adjusted dose according to trough levels >10 g/ml and also AUC/MIC >400 (Table 1).

**Table 1:** Daily dose medians, trough and PK/PD data

Parameter	Initial dose	Adjusted dose	*P *value
Daily dose (mg/kg/day)	28.6	42.9	0.01
Trough levels (μg/ml)	7.1(3.6 to 13.3)	16.0 (12.0 to 23.3)	<0.001
AUC/MIC (MIC: 1 mg/l)	436 (248 to 659)	648 (467 to 942)	0.02

Data presented as median (25 to 75% percentile).

## Conclusion

The initial dose recommended for vancomycin must be increased according to trough levels and also AUC/MIC to achieve the PK/PD target in burn patients with preserved renal function.
